# Acute and Long-Term Toxicity after Planned Intraoperative Boost and Whole Breast Irradiation in High-Risk Patients with Breast Cancer—Results from the Targeted Intraoperative Radiotherapy Boost Quality Registry (TARGIT BQR)

**DOI:** 10.3390/cancers16112067

**Published:** 2024-05-30

**Authors:** Lukas Goerdt, Robert Schnaubelt, Uta Kraus-Tiefenbacher, Viktoria Brück, Lelia Bauer, Stefan Dinges, Albert von der Assen, Heidrun Meye, Christina Kaiser, Christel Weiss, Sven Clausen, Frank Schneider, Yasser Abo-Madyan, Katharina Fleckenstein, Sebastian Berlit, Benjamin Tuschy, Marc Sütterlin, Frederik Wenz, Elena Sperk

**Affiliations:** 1 Department of Gynecology and Obstetrics, University Medical Center Mannheim, Medical Faculty Mannheim, Heidelberg University, 68167 Mannheim, Germany; lukas.goerdt@umm.de (L.G.);; 2 Radiation Oncology, MVZ Rheinland Klinikum Neuss, 41462 Neuss, Germany; 3 Department of Radiation Oncology, Krankenhaus Nordwest, 60488 Frankfurt am Main, Germany; 4 Breast Center, Asklepios Klinik Barmbek, 22307 Hamburg, Germany; 5 Breast Center, GRN Klinik Weinheim, 69469 Weinheim, Germany; 6 Department of Radiation Oncology, Städtisches Klinikum Lüneburg, 21339 Lüneburg, Germany; 7 Breast Center, Department of Senology, Franziskus Hospital Harderberg—Niels Stensen Kliniken, 49124 Georgsmarienhütte, Germany; 8 Department of Radiation Oncology, MVZ Gesundheit Nordhessen, 34125 Kassel, Germany; 9 University Medical Center Bonn, Medical Faculty Bonn, Bonn University, 53113 Bonn, Germany; 10 Department of Medical Biometry, University Medical Center Mannheim, Medical Faculty Mannheim, Heidelberg University, 68167 Mannheim, Germany; 11 Department of Radiation Oncology, University Medical Center Mannheim, Medical Faculty Mannheim, Heidelberg University, 68167 Mannheim, Germany; 12 University Hospital Freiburg, 79106 Freiburg, Germany; 13 Mannheim Cancer Center, University Medical Center Mannheim, Medical Faculty Mannheim, Heidelberg University, 68167 Mannheim, Germany

**Keywords:** IORT, intraoperative radiotherapy, boost, breast cancer, high risk, toxicity, fibrosis, whole breast irradiation

## Abstract

**Simple Summary:**

This multicenter study (n = 1133, inclusion criteria: 3.5 cm maximum tumor size and preoperative indication for a boost) provides detailed data on acute and long-term toxicities in breast cancer patients undergoing breast-conserving surgery combined with an anticipated intraoperative boost with low-energy X-rays followed by whole breast irradiation. Toxicity assessments, based on LENT SOMA criteria, were performed annually up to 10 years of follow-up. IORT boost was completed in 90% and EBRT in 97% of cases. No grade 3 or 4 acute toxicities were observed, with mild acute side effects reported in a small proportion of patients. Chronic toxicities were seen in 16.2% of patients with teleangiectasia, 14.3% with grade ≥ 2 fibrosis, 3.4% with grade ≥ 2 pain, and 1.1% with hyperpigmentation. The results show that the therapy is safe and feasible in terms of toxicity and confirms intraoperative boost as a standard method of boost application in a large prospective cohort.

**Abstract:**

In the context of breast cancer treatment optimization, this study prospectively examines the feasibility and outcomes of utilizing intraoperative radiotherapy (IORT) as a boost in combination with standard external beam radiotherapy (EBRT) for high-risk patients. Different guidelines recommend such a tumor bed boost in addition to whole breast irradiation with EBRT for patients with risk factors for local breast cancer recurrence. The TARGIT BQR (NCT01440010) is a prospective, multicenter registry study aimed at ensuring the quality of clinical outcomes. It provides, for the first time, data from a large cohort with a detailed assessment of acute and long-term toxicity following an IORT boost using low-energy X-rays. Inclusion criteria encompassed tumors up to 3.5 cm in size and preoperative indications for a boost. The IORT boost, administered immediately after tumor resection, delivered a single dose of 20 Gy. EBRT and systemic therapy adhered to local tumor board recommendations. Follow-up for toxicity assessment (LENT SOMA criteria: fibrosis, teleangiectasia, retraction, pain, breast edema, lymphedema, hyperpigmentation, ulceration) took place before surgery, 6 weeks to 90 days after EBRT, 6 months after IORT, and then annually using standardized case report forms (CRFs). Between 2011 and 2020, 1133 patients from 10 centers were preoperatively enrolled. The planned IORT boost was conducted in 90%, and EBRT in 97% of cases. Median follow-up was 32 months (range 1–120, 20.4% dropped out), with a median age of 61 years (range 30–90). No acute grade 3 or 4 toxicities were observed. Acute side effects included erythema grade 1 or 2 in 4.4%, palpable seroma in 9.1%, punctured seroma in 0.3%, and wound healing disorders in 2.1%. Overall, chronic teleangiectasia of any grade occurred in 16.2%, fibrosis grade ≥ 2 in 14.3%, pain grade ≥ 2 in 3.4%, and hyperpigmentation in 1.1%. In conclusion, a tumor bed boost through IORT using low-energy X-rays is a swift and feasible method that demonstrates low rates in terms of acute or long-term toxicity profiles in combination with whole breast irradiation.

## 1. Introduction

Breast cancer remains a significant global health challenge, standing as the most prevalent malignancy among women and contributing to substantial mortality rates [1,2]. Despite advancements in prevention and screening, the cornerstone of breast cancer therapy still encompasses breast-conserving surgery, subsequent radiotherapy, and adjuvant systemic treatment [3,4,5,6]. Categorizing patients into high-risk and low-risk groups is integral for tailoring treatment, with the administration of a boost being a crucial component for high-risk cases. High-risk patients are patients with an age of less than 50 years or patients with an age of 51 years and one the following components: grading 3, Her2neu positive tumors, triple negative tumors, T2 or other local defined high-grade indicators [7,8,9]. Various techniques are available for boost delivery, including brachytherapy, sequential external beam radiotherapy (EBRT), simultaneous integrated boost (SIB) during whole breast irradiation (WBI), and intraoperative radiotherapy (IORT) with low-kV X-rays or electrons [10,11,12,13,14,15,16,17].

Among these techniques, IORT with low-kV X-rays stands out for its potential to reduce overall treatment time while minimizing radiation exposure to nearby vital organs [18,19,20,21]. However, like conventional radiotherapy approaches, IORT can be accompanied by certain side effects, notably fibrosis, seroma and other concerns. The occurrence of these side effects can influence patient compliance, treatment adherence, and in the end, overall oncological outcomes [22,23,24,25].

The TARGIT BQR (Targeted Intraoperative Radiotherapy Boost Quality Registry) phase IV study (NCT01440010) aims to comprehensively explore the acute and long-term side effect profile of IORT with low-kV X-rays, while also considering its oncological and cosmetic implications. Notably, this study is the first to present prospective and detailed data on acute and long-term toxicities within a representative patient cohort in a registry setting, thus contributing invaluable insights into the quality assurance of IORT.

This paper primarily focuses on the analysis of side effects, highlighting the paramount importance of treatment-related toxicities to ensure optimal conditions for patients and caregivers to make evidence-based treatment choices.

## 2. Materials and Methods

**Figure 1:**
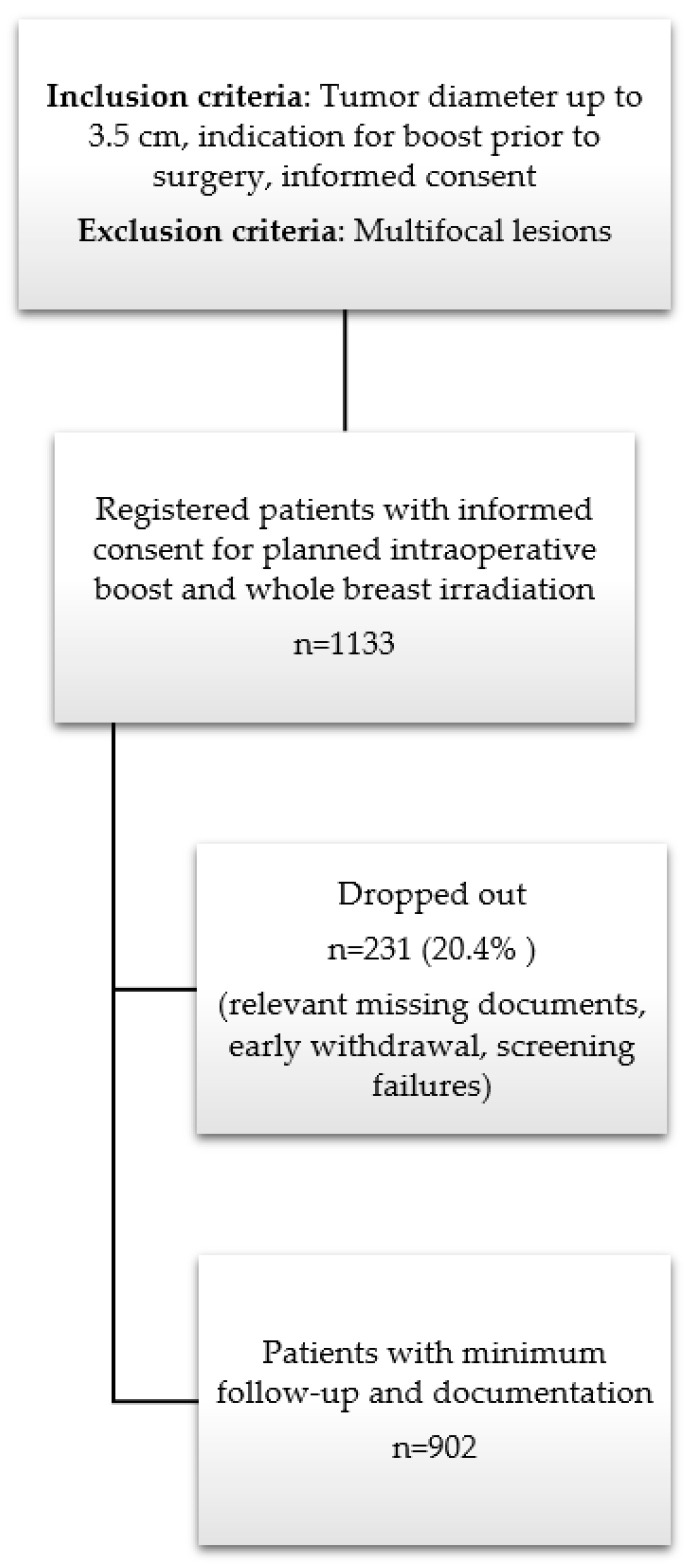
Study design and patient recruitment within TARGIT BQR.

TARGIT BQR started recruitment in 2011 after approval by the local ethics committee (2011-319N-MA) and registration in clinicaltrials.gov (NCT01440010). Ten centers participated in this multicenter national registry in Germany. As this is a phase IV trial and IORT is one option for boost delivery in the German guidelines, no specific protocol is available. Patients were recruited based on predefined inclusion and exclusion criteria (Figure 1) [7,9,26]. All patients gave their informed consent prior to surgery and were planned for an anticipated boost immediately after tumor resection. Additionally, all patients were planned for whole breast radiotherapy with external beam radiation. The detailed procedure of IORT with low-kV X-rays (12–20 Gy as a single fraction with the INTRABEAM^®^ system; Zeiss Meditec AG, Oberkochen, Germany) was already described elsewhere and EBRT was given according to standard of care procedures with conventional or hypofractioned schedules [25,27]. Systemic treatment was given according to local recommendations after discussion within the multidisciplinary tumor board. When chemotherapy was indicated, it could be given prior to surgery (neoadjuvant) or between IORT and EBRT. The recommended interval between IORT and EBRT was at least 35 days [25]. Follow-up was conducted 6 weeks after the end of EBRT, 6 months after IORT and yearly thereafter using paper-based case report forms (CRFs). Toxicity was recorded based on LENT SOMA scale [28,29]. For cumulative toxicity rates, grade 2 fibrosis, breast edema, lymphedema, hyperpigmentation, retraction, and pain were documented if they occurred at least 3 times during the follow-up period. Teleangiectasia and ulceration, irrespective of their severity, were recorded as single occurrences. Data on acute and long-term toxicity were recorded. Simple frequencies, relative and absolute values, and cumulative rates were calculated. Cumulative rates were calculated based on the Kaplan–Meier method (SAS, release 9.3, SAS Institute, Cary, NC, USA and SPSS, Version 27, IBM, Armonk, NY, USA). This is an intention-to-treat analysis of the whole cohort. All data were collected at the study center in Mannheim. The registry closed recruitment in December 2020.

## 3. Results

In total, 1133 patients were registered, of whom 10 patients had both-sided carcinomas at the same time (1143 carcinomas included). Of those 1133 patients, 231 patients (20.4%) dropped out due to failures in informed consent documents, relevant missing data, screening failures or early withdrawals without follow-up data. Table 1 shows the detailed numbers from the different centers and for the whole cohort. Most patients were recruited (n = 561 (49.5%)) from two centers. Not all patients had data at all follow-up slots and for all categories.

### 3.1. Patient and Tumor Characteristics

Table 2 gives an overview of the main patient and tumor characteristics of the 902 patients with 910 carcinomas. Breast volume was known from 469 patients and was 867 mL in median with a maximum of 922 mL and a minimum of 200 mL. Seroma volumes were reported in only 293 patients and ranged between 0 and 347 mL.

### 3.2. Treatment Characteristics

Table 3 gives an overview of the treatment details. In most cases, IORT was performed as planned (90%). In 22 cases, IORT could not be performed because the wound cavity was too large. In 53 cases, the skin distance was too small; in one case, there was an organizational problem; and in three cases, a technical problem occurred. In 13 cases, the reason was unknown. One-third of patients had additional chemotherapy, mostly given between IORT and EBRT. EBRT was given in 97% of patients.

### 3.3. Acute Toxcity

Acute toxicity was assessed during the first 90 days after surgery/IORT. No grade 3 to 4 toxicity occurred. Table 4 shows acute toxicity outcomes.

### 3.4. Late Toxicity

No grade 5 toxicity occurred during the whole follow-up up of ten years. Toxicity after surgery and IORT boost (between IORT boost and start of EBRT) was very low. Most toxicities appeared after finishing whole breast irradiation. Table 5 and Table 6 show detailed late toxicity data according to the assessments at each time point during the whole follow-up time of 10 years.

**Figure 2:**
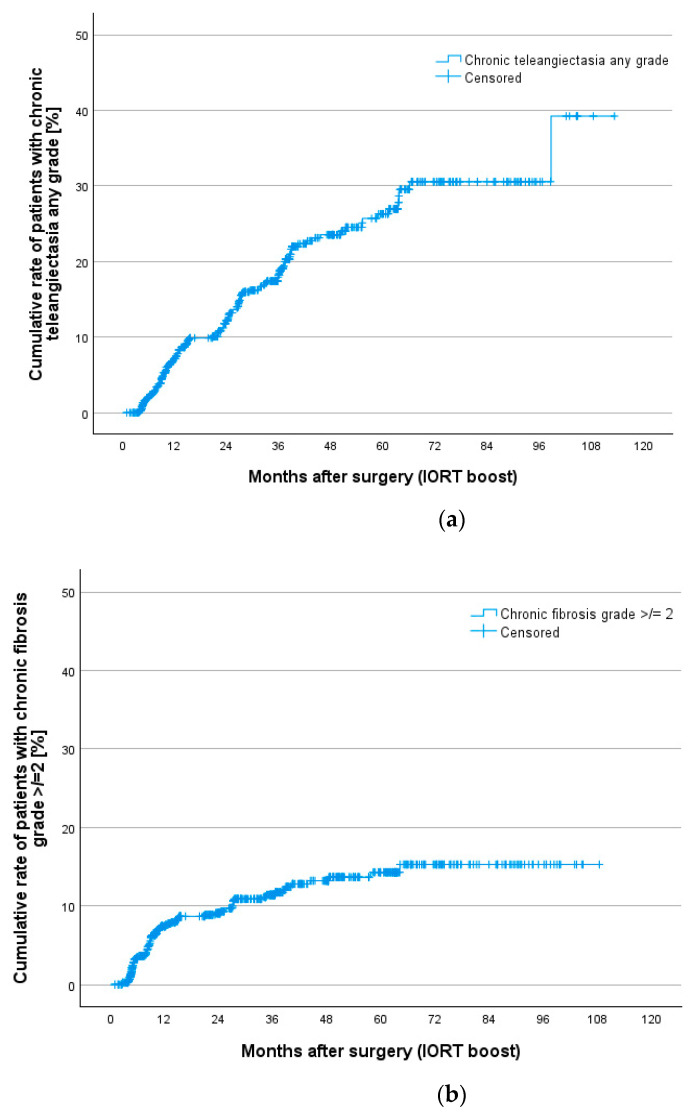

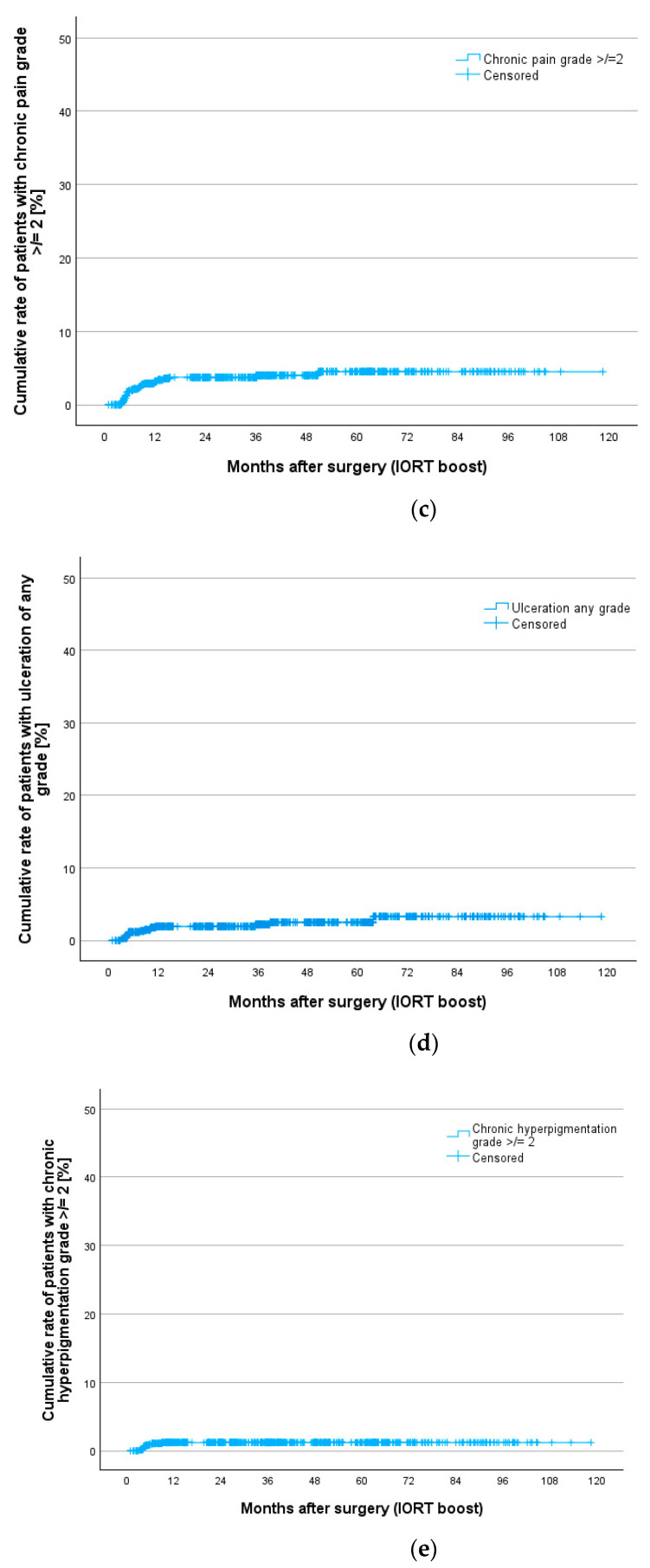
Cumulative rates for chronic toxicities after IORT boost and whole breast irradiation in the TARGIT BQR cohort according to LENT SOMA scale for (**a**) teleangiectasia, (**b**) fibrosis, (**c**) pain, (**d**) ulceration and (**e**) hyperpigmentation.

Additionally, cumulative rates for all toxicities were calculated to show chronic toxicities presenting at least 3 times during the whole follow-up. This gives us better information on persistent clinically relevant findings. Table 7 gives an overview of chronic toxicity rates at 12, 24, 36, 60 and 84 months according to Kaplan–Meier estimation. Nearly all toxicities occur during the first 5 years of follow-up. Figure 2 shows Kaplan–Meier curves for teleangiectasia, fibrosis, pain, ulceration and hyperpigmentation. Teleangiectasia with grade 1 or higher was the most seen toxicity followed by fibrosis grade 2 or higher. Chronic pain grade 2 or higher occurred in 4.5% after 5 and 7 years.

## 4. Discussion

The TARGIT BQR study is the largest longitudinal multicenter study with 1133 registered patients from 10 centers in Germany providing prospective data on IORT boost with low-kV X-rays in combination with whole breast irradiation. With a planned follow-up of up to 10 years, this study recorded detailed acute and long-term toxicity data, which are presented here. Despite the combination of a high single dose of 20 Gy given immediately after tumor resection and standard doses of 40–50 Gy with EBRT for whole breast irradiation, no grade 3 or 4 acute toxicities were seen. Wound healing disorders were also low with 2.1%, consistent with rates typically observed without the inclusion of IORT. Long-term toxicities were also in range with known data after EBRT. Overall, the most seen cumulative rates of chronic toxicities were teleangiectasia (16.2%), fibrosis grade 2 or higher (9.9%) and pain grade 2 or higher (3.4%). All other toxicities like ulceration, hyperpigmentation, breast edema, retraction and lymphedema occurred in less than 2%.

One of the oldest and largest studies regarding effectiveness and toxicity of adding a boost to whole breast irradiation was the EORTC boost vs. no boost trial [21]. Here, a total of 2661 patients with an EBRT boost after whole breast irradiation and 2657 without boost were followed with a median of 17.2 years. Fibrosis was the only toxicity examined in this study regarding possible side effects. The cumulative rate of moderate-to-severe (grade 2 to 3) fibrosis was seen in 15.0% at 20 years in the no-boost group and in 30.4% in the boost group, which was significantly higher with a *p*-value of <0.0001. As this was a completely other technique and also time point (after EBRT), we also have to compare our results to the same or similar techniques and also to modern techniques like integrated boost schemes. Our results match with retrospective data for IORT boost with low-kV X-rays provided by Blank et al. and Wenz et al. in 2010, which consistently showed good side effect profiles of IORT boost. The most seen side effects in these publications were the same as seen in TARGIT BQR: fibrosis (37.9% and 22.7%), teleangiectasia (12.1% and 3.9%), and pain (22.4% and 0.6%). Other toxicities occurred with a very small percentage or not at all [24,25]. Another study by Pez et al. provided results similar to those of Blank et al. and Wenz et al., but with a significantly larger study population (n = 400) and a significantly longer follow-up period of 78 months. Again, fibrosis (19.1%), teleangiectasia (10.0%) and pain (8.6%) were the main side effects [22].

A study by Wang et al. from 2022 showed an extremely low side effect profile in an equally large cohort of a total of n = 451 patients and a long follow-up of almost 65 months, in which none of the side effects occurred in more than 1.3% of the patients [23]. Table 8 gives an overview of studies using IORT boost using low-kV X-rays.

Other techniques to provide a boost are IORT with electrons (IOERT) and brachytherapy. Studies using brachytherapy recording at least parts of the toxicity profile used in our study show good results [32]. In studies using IOERT, mostly a mix of acute and late toxicities is reported without detailed information on certain toxicities. For example, Ciabattoni et al. show low rates of higher-grade toxicities without any further information [33].

In the study by Van Hulle et al., a simultaneous integrated boost (SIB) was given and compared with a sequential EBRT boost (SEB), which is the same as that used in the EORTC boost trial. Here, comparable results were shown without the occurrence of grade 3 or 4 toxicities, whereby the SEB compared to the SIB showed slightly increased rates of all parameters, except for hyperpigmentation [34]. The study by Palumbo et al. evaluated sequential hypofractionated boost in one institution in 219 patients and showed no negative effects of hypofractionated boost regarding toxicity and cosmesis in a follow-up of up to 12 months [35]. Acute toxicities after SIB and SEB were reported from the MINT trial by Krug et al., showing a difference between grade 2 and 3 radiation dermatitis in favor of the SEB, whereas breast and chest wall pain occurred significantly more in the SEB group [36]. Hörner-Rieber et al. showed long-term results from the same phase III trial and showed no differences for late toxicities according to LENT SOMA between SIB und SEB [37]. Table 9 gives an overview of boost studies using brachytherapy, IOERT and the SIB technique. A comparison of results should be performed carefully as different scales (LENT SOMA, CTC) were used and different perceptions on how to show data were applied. Another caveat is that toxicity rates might be underestimated in retrospective trials by the nature of the retrospective study design. In addition, different views on which manifestations of toxicities are clinically relevant can lead to different results in similar data sets. This is specifically important in our study for teleangiectasia rates as we included all grades (≥1) of teleangiectasia as clinically relevant. This is different compared to most other studies where usually grade ≥2 is used as clinically relevant and might implicate a higher rate in our study compared to other studies.

The potpourri of boost techniques gives patients and caregivers many different options. A limitation of IORT boost compared to other techniques may be the very limited volume of irradiated tissue, making an assessment of the tumor cavity during surgery crucial. Also, the high single dose needs to be carefully prescribed with a certain distance to the skin [38]. On the other hand, IORT boost with kV X-rays provides immediate irradiation and very good volume definition. It avoids the need for surgical clips, which are highly recommended for postoperative boost techniques [39]. Patients can undergo oncoplastic surgery immediately after IORT of the resection cavity with kV X-ray IORT boost. As volume definition for a postoperative boost is often difficult to perform in the setting of oncoplastic lumpectomy and may need to be omitted, intraoperative boost may provide a treatment solution for this specific scenario.

**Table 9:** Main studies using other techniques for boost and reporting toxicities. BT = Brachytherapy, IOERT = Intraoperative electron boost, WBI = Whole breast irradiation, IMRT = Intensity modulated radiotherapy.

Method	Brachytherapy	IOERT		Simultaneous Integrated Boost (SIB) vs. Sequential EBRT Boost (SEB)	
author	Chichel et al. [32]	Ciabattoni et al. [40]	Fastner et al. [41]	Van Hulle et al. [34]	Krug et al./Hoerner-Rieber et al. [36,37]
year	2022	2022	2020	2021	2020
follow-up (median months)	95	57	45	24	61
Patients (n)	57	797	583	76	446
total dose (Gy) (dose for fraction) and technique	BT Boost 10 Gy WBI 42.5–50.0 Gy	IOERT dose 9–12 Gy WBI dose 50 Gy (75.5%) 40.5/42.56 Gy (23.7%)	IOERT dose 11.1 Gy WBI 40.5 Gy in 15 fractions	WBI: 40.05 Gy in 15 fractions SIB: 46.8 or 49.95 Gy (15 fractions) SEB: 10 Gy(4 fractions)or 14.88 Gy (6 fractions)	IMRT: 50.4 Gy SIB: 64.4 Gy (28 fractions)/ SEB: 16 Gy (8 fractions)
fibrosis	33.3%	Seroma requiring surgical drainage (22.3%) Surgical wound infection (0.12%) Fibrosis G1–G3 (42.2%) Teleangiectasia G1–G3 (0.13%)	Late toxicities G3: Pain: 0.2% Breastedema: 0.07% Fibrosis: 0.7% Teleangiectasia: 0.4% Retraction: 1.4%	Out of tumour bed (G1–G2) SIB: 13%/SEB12.7% In tumour bed (G1–G2) SIB: 7.2%/SEB: 9.1%	Radiation Dermatitis G2–G3: SIB: 32.6% SEB: 22.4% Breast/chest wall pain G1–G2: SIB: 15% SEB: 19.1%
teleangiectasia	4%			(G1–G2) SIB: 5.8%/SEB: 7.3%	
hyperpigmentation				(G1–G2) SIB: 22.1%/SEB: 17.6%	
breast edema				(G1–G2) SIB: 4.3%/SEB: 7.3%	
lymph edema				-	
retraction				(G1–G2) SIB: 25.7%/SEB: 28.6%	
ulceration				-	
pain				(G1–G2) SIB: 0%/SEB: 7.3%	

### Limitations and Strengths of the Study

Although this study is a multicenter study, it only includes German centers, so the applicability of the results to other population groups might be limited. Furthermore, the patients in our study were mostly irradiated with 50 Gy and normofractionated schedules, although the standard today is hypofractionation. Still, the data regarding toxicities can be compared due to the known and comparable toxicity profiles after hypofractionation compared to normofractionation [42].

Another limitation of this study is the limited cohort of patients for whom a 5-year follow-up examination is available. This could be a result of the fact that TARGIT BQR is an investigator-initiated trial, financed purely from institute funds and without payment to the centers. Under these circumstances, the amount of data available is actually considerable.

The TARGIT BQR study adds new data to the field by providing detailed toxicity data over a long follow-up period in a large prospective cohort. This might be important as therapy-related side effects have an impact on the quality of life both directly and indirectly, for example, via their influence on the cosmetic result [43]. Recent data have shown that IORT boost with low-kV X-rays also results in good cosmetic outcomes in the long run [44]. It is well researched that an increased quality of life has a positive effect on the oncological outcome [45,46]. Therefore, the current results for toxicity and cosmetic outcome from the TARGIT BQR are encouraging.

## 5. Conclusions

IORT with low-kV X-rays was performed in 90% of the prospectively enrolled patients and has a low spectrum of acute side effects and low rates of clinically relevant late toxicity. It therefore fulfills high-quality standards and is safe and feasible.

## Figures and Tables

**Table 1:** Number of recruited patients and breast cancers per center and for the whole cohort within TARGIT BQR.

Participating Site	Registered Patients	Dropped Out Patients	Treated Patients in Current Analysis	Both-Sided Breast Cancer at Diagnosis	Registered Breast Cancers	Dropped Out Cancers	Treated Cancers in Current Analysis
All	1133	231	902	10	1143	233	910
Neuss	298	32	266	4	302	33	269
Mannheim	263	28	235	2	265	28	237
Frankfurt	180	66	114	0	180	66	114
Lich	166	48	118	0	166	48	118
Weinheim	148	32	116	4	152	33	119
Lüneburg	37	4	33	0	37	4	33
Osnabrück	28	21	7	0	28	21	7
Kassel	6	0	6	0	6	0	6
Bonn	6	0	6	0	6	0	6
Pinneberg	1	0	1	0	1	0	1

**Table 2:** Patient and tumor characteristics of the TARGIT BQR cohort.

General	
Number of analyzed patients	n = 902 (100%)
Age at surgery (years, median (min–max.))	61 (30–90)
Death	n = 16 (1.8%)
Total number of carcinomas	910
**Tumor characteristics**	**Number of patients (%)**
Localization	
Left breast	452 (49.70%)
Right breast	450 (49.50%)
Both sides	8 (0.90%)
Upper outer quadrant	495 (54.40%)
Upper inner quadrant	167 (18.40%)
Lower outer quadrant	113 (12.41%)
Lower inner quadrant	60 (6.60%)
Upper outer + Upper inner quadrant	31 (3.41%)
Lower outer + Lower inner quadrant	4 (0.44%)
Central/retromamillary	12 (1.32%)
Upper outer + Lower outer quadrant	14 (1.54%)
Upper inner + Lower inner quadrant	10 (1.10%)
Unknown	4 (0.44%)
Family history (breast cancer)	
Positive	237 (26.04%)
Negative	665 (73.10%)
Histology	
No special type (NST)	687 (75.50%)
Invasive-lobular	86 (9.50%)
Other	54 (5.93%)
NST + lobular	4 (0.11%)
Unknown	79 (8.70%)
T *	
0	11 (1.21%)
1	627 (68.90%)
2	174 (19.12%)
3	6 (0.70%)
Unknown	92 (10.10%)
N *	
0	654 (71.90%)
1	155 (17.03%)
2	16 (1.80%)
3	5 (0.60%)
Unknown	80 (8.80%)
M *	
0	778 (85.50%)
1	5 (0.60%)
Not specified	3 (0.33%)
Unknown	124 (13.63%)
G *	
0	5 (0.55%)
1	178 (19.60%)
2	446 (49.01%)
3	192 (21.10%)
Unknown	89 (9.80%)
L-Status *	
0	713 (78.40%)
1	93 (10.22%)
Unknown	104 (11.43%)
V-Status *	
0	752 (82.64%)
1	7 (0.80%)
Unknown	151 (16.60%)
R-Status *	
0	799 (87.80%)
1	22 (2.42%)
Unknown	89 (9.80%)
Estrogen receptor	
Positive	724 (79.60%)
Negative	106 (11.70%)
Unknown	80 (8.80%)
Progesterone receptor	
Positive	668 (73.41%)
Negative	159 (17.50%)
Unknown	83 (9.12%)
HER2neu	
Positive	86 (9.50%)
Negative	748 (82.20%)
Unknown	76 (8.10%)
Tumor size	
>3.5 cm	0 (0.00%)
≤3.5 cm	907 (99.70%)
Unknown	3 (0.33%)

* T = tumor size according to the TNM-Classification, N = involved lymph nodes according to the TNM-Classification, M = distant metastasis according to the TNM-Classification, G = Grading according to the TNM-Classification, L-Status = invasion into lymphatic vessels according to the TNM-Classification, V-Status = invasion into veins according to the TNM-Classification, R-Status = resection status according to the TNM-Classification [30,31].

**Table 3:** Treatment details of the TARGIT BQR cohort.

Treatment Details	Median
IORT (intraoperative radiotherapy) dose (Gy)	20
Irradiation time (minutes)	24
Applicator size (mm)	35
EBRT (external beam radiotherapy) total dose (Gy)	50.4
EBRT single fraction (Gy)	1.8
**Treatment details**	**Number of patients (%)**
Chemotherapy	
Yes	293 (32.20%)
No	541 (59.50%)
Unknown	76 (8.40%)
Neoadjuvant	
Yes	153 (16.81%)
No	681 (74.84%)
Unknown	76 (8.40%)
adjuvant	
Yes	152 (16.70%)
No	675 (74.20%)
unknown	83 (9.12%)
Endocrine therapy regimen	
Tamoxifen	
Yes	317 (34.84%)
No	518 (56.92%)
unknown	75 (8.24%)
Aromatase inhibitors (AI)	
Yes	173 (19.01%)
No	662 (72.80%)
unknown	75 (8.24%)
Switch (Tamoxifen followed by AI)	
Yes	225 (24.73%)
No	610 (67.03%)
unknown	75 (8.24%)
Tamoxifen + GnRH analogue	
Yes	11 (1.21%)
No	824 (90.60%)
unknown	75 (8.24%)
Herceptin	
Yes	58 (6.40%)
No	777 (85.40%)
unknown	75 (8.24%)
Re-resection	
Due to close margin	19 (2.10%)
Due to Toxicities	2 (0.22%)

**Table 4:** Acute toxicities up to 90 days after surgery, IORT boost and whole breast irradiation within the TARGIT BQR cohort.

Acute Toxicity	Whole Cohort n = 910
Erythema before EBRT n (%)	40 (4.4)
G1: n (%)	29 (72.5)
G2: n (%)	11 (27.5)
Palpable seroma n (%)	83 (9.1)
Seroma puncture > 3x n (%)	3 (0.3)
Wound healing disorder n (%)	19 (2.1)

**Table 5:** Longitudinal assessment of late toxicities according to LENT SOMA with detailed data at different timepoints for fibrosis, pain, ulceration and retraction.

Follow-Up	Fibrosis [n (%)]				Pain [n (%)]				Ulceration [n (%)]				Retraction [n (%)]				
	°0	°1	°2	°3	°0	°1	°2	°3	°0	°1	°2	°3	°0	°1	°2	°3	°4
before EBRT	438 (55.7)	246 (31.3)	101 (12.9)	1 (0.1)	530 (67.4)	216 (27.5)	38 (4.8)	2 (0.3)	782 (99.5)	4 (0.5)	0 (0.0)	0 (0.0)	737 (93.9)	48 (6.1)	0 (0.0)	0 (0.0)	0 (0.0)
1–6 weeks after EBRT	263 (39.9)	264 (40.1)	127 (19.3)	5 (0.8)	365 (55.3)	233 (35.3)	58 (8.8)	4 (0.6)	642 (97.6)	13 (2.0)	2 (0.3)	1 (0.2)	578 (88.1)	75 (11.4)	3 (0.5)	0 (0.0)	0 (0.0)
6 months	147 (36.0)	162 (39.7)	94 (23.0)	5 (1.2)	219 (53.6)	134 (32.8)	50 (12.2)	6 (1.5)	405 (99.3)	1 (0.3)	0 (0.0)	2 (0.5)	328 (80.6)	71 (17.4)	7 (1.7)	1 (0.3)	0 (0.0)
1 year	177 (32.4)	251 (46.0)	115 (21.1)	3 (0.6)	323 (59.4)	168 (30.9)	47 (8.6)	6 (1.1)	541 (99.5)	2 (0.4)	1 (0.2)	0 (0.0)	416 (76.3)	123 (22.6)	6 (1.1)	0 (0.0)	0 (0.0)
2 years	170 (37.0)	198 (43.0)	84 (18.3)	8 (1.7)	303 (65.4)	131 (28.3)	24 (5.2)	5 (1.1)	459 (99.8)	1 (0.2)	0 (0.0)	0 (0.0)	330 (71.7)	119 (25.9)	10 (2.2)	1 (0.2)	0 (0.0)
3 years	130 (37.8)	142 (41.3)	65 (18.9)	7 (2.0)	243 (68.6)	87 (24.6)	20 (5.7)	4 (1.1)	341 (99.1)	1 (0.3)	1 (0.3)	1 (0.3)	234 (68.0)	97 (28.2)	11 (3.2)	1 (0.3)	1 (0.3)
4 years	99 (39.8)	83 (33.3)	57 (22.9)	10 (4.0)	172 (66.7)	62 (24.0)	23 (8.9)	1 (0.4)	248 (100)	0 (0.0)	0 (0.0)	0 (0.0)	150 (60.2)	88 (35.3)	9 (3.6)	1 (0.4)	1 (0.4)
5 years	45 (27.3)	63 (38.2)	51 (30.9)	6 (3.6)	98 (58.0)	55 (32.5)	14 (8.3)	2 (1.2)	163 (99.4)	0 (0.0)	1 (0.6)	0 (0.0)	94 (57.0)	64 (38.8)	7 (4.2)	0 (0.0)	0 (0.0)
6 years	21 (29.6)	29 (40.9)	18 (25.4)	3 (4.2)	45 (57.7)	23 (29.5)	7 (9.0)	3 (3.9)	68 (100)	0 (0.0)	0 (0.0)	0 (0.0)	42 (61.8)	22 (32.4)	3 (4.4)	0 (0.0)	1 (1.5)
7 years	11 (28.2)	14 (35.9)	12 (30.8)	2 (5.1)	29 (61.7)	13 (27.7)	5 (10.6)	0 (0.0)	37 (100)	0 (0.0)	0 (0.0)	0 (0.0)	26 (78.8)	7 (21.2)	0 (0.0)	0 (0.0)	0 (0.0)
8 years	1 (6.7)	5 (33.3)	7 (46.7)	2 (13.3)	6 (42.9)	3 (21.4)	5 (35.7)	0 (0.0)	12 (100)	0 (0.0)	0 (0.0)	0 (0.0)	6 (46.2)	6 (46.2)	1 (7.7)	0 (0.0)	0 (0.0)
9 years	2 (28.6)	5 (71.4)	0 (0.0)	0 (0.0)	6 (75.0)	2 (25.0)	0 (0.0)	0 (0.0)	7 (100)	0 (0.0)	0 (0.0)	0 (0.0)	3 (42.9)	3 (42.9)	1 (14.3)	0 (0.0)	0 (0.0)
10 years	0 (0.0)	0 (0.0)	1 (100)	0 (0.0)	0 (0.0)	0 (0.0)	1 (100)	0 (0.0)	1 (100)	0 (0.0)	0 (0.0)	0 (0.0)	0 (0.0)	1 (100)	0 (0.0)	0 (0.0)	0 (0.0)

**Table 6:** Longitudinal assessment of late toxicities according to LENT SOMA with detailed data at different timepoints for teleangiectasia, lymphedema, breast edema and hyperpigmentation.

Follow-Up	Teleangiectasia [n (%)]				Lymphedema [n (%)]				Breast Edema [n (%)]				Hyperpigmentation [n (%)]		
	°0	°1	°2	°3	°0	°1	°2	°3	°0	°1	°2	°3	°0	°1	°2
before EBRT	780 (99.2)	5 (0.6)	1 (0.1)	0 (0.0)	762 (97.1)	21 (2.7)	1 (0.1)	1 (0.1)	702 (89.1)	74 (9.4)	12 (1.5)	0 (0.0)	719 (91.6)	58 (7.4)	8 (1.0)
1–6 weeks after EBRT	636 (96.2)	21 (3.2)	3 (0.5)	1 (0.2)	620 (93.9)	38 (5.8)	2 (0.3)	0 (0.0)	506 (76.8)	125 (19.0)	28 (4.3)	0 (0.0)	334 (50.8)	273 (41.6)	50 (7.6)
6 months	381 (94.1)	17 (4.2)	5 (1.2)	2 (0.5)	373 (91.7)	32 (7.9)	2 (0.5)	0 (0.0)	300 (73.7)	80 (19.7)	27 (6.6)	0 (0.0)	239 (58.7)	136 (33.4)	32 (7.9)
1 year	507 (92.9)	26 (4.8)	11 (2.0)	2 (0.4)	510 (93.8)	29 (5.3)	4 (0.7)	1 (0.2)	401 (73.7)	103 (18.9)	38 (7.0)	2 (0.4)	376 (69.4)	132 (24.4)	34 (6.3)
2 years	419 (91.1)	24 (5.2)	15 (3.3)	2 (0.4)	432 (93.9)	26 (5.7)	2 (0.4)	0 (0.0)	388 (84.4)	57 (12.4)	13 (2.8)	2 (0.4)	394 (85.7)	55 (12.0)	11 (2.4)
3 years	301 (87.8)	19 (5.5)	19 (5.5)	4 (1.2)	326 (94.0)	21 (6.1)	0 (0.0)	0 (0.0)	305 (88.9)	32 (9.3)	6 (1.8)	0 (0.0)	309 (89.8)	25 (7.3)	10 (2.9)
4 years	211 (84.4)	18 (7.2)	17 (6.8)	4 (1.6)	228 (91.6)	18 (7.2)	3 (1.2)	0 (0.0)	212 (84.8)	30 (12.0)	8 (3.2)	0 (0.0)	235 (92.1)	11 (4.4)	2 (0.8)
5 years	131 (80.4)	18 (11.0)	11 (6.8)	3 (1.8)	154 (93.3)	10 (6.1)	1 (0.6)	0 (0.0)	146 (89.6)	13 (8.0)	3 (1.8)	1 (0.6)	152 (92.1)	9 (5.5)	4 (2.4)
6 years	56 (84.9)	5 (7.6)	4 (6.1)	1 (1.5)	64 (90.1)	6 (8.5)	1 (1.4)	0 (0.0)	58 (84.1)	7 (10.1)	3 (4.4)	1 (1.5)	64 (94.1)	1 (1.5)	3 (4.4)
7 years	29 (82.9)	3 (8.6)	3 (8.6)	0 (0.0)	37 (92.5)	3 (7.5)	0 (0.0)	0 (0.0)	33 (86.8)	4 (10.5)	1 (2.6)	0 (0.0)	34 (94.4)	2 (5.6)	0 (0.0)
8 years	11 (84.6)	1 (7.7)	0 (0.0)	1 (7.7)	12 (100)	0 (0.0)	0 (0.0)	0 (0.0)	10 (76.9)	2 (15.4)	1 (7.7)	0 (0.0)	12 (100)	0 (0.0)	0 (0.0)
9 years	6 (85.7)	0 (0.0)	1 (14.3)	0 (0.0)	6 (85.7)	1 (14.3)	0 (0.0)	0 (0.0)	5 (71.4)	2 (28.6)	0 (0.0)	0 (0.0)	6 (85.7)	1 (14.3)	0 (0.0)
10 years	0 (0.0)	0 (0.0)	1 (100)	0 (0.0)	1 (100)	0 (0.0)	0 (0.0)	0 (0.0)	1 (100)	0 (0.0)	0 (0.0)	0 (0.0)	1 (100)	0 (0.0)	0 (0.0)

**Table 7:** Cumulative chronic toxicity rates according to LENT SOMA at different time points and overall occurrence. Toxicities were rated as chronic after presenting at least 3 times during the whole follow-up.

Time after Surgery (IORT Boost)	12 Months [%]	24 Months [%]	36 Months [%]	60 Months [%]	84 Months [%]	Overall [Patients; %]
Teleangiectasia any grade	7.1	12.2	18.2	26.3	30.6	132/813; 16.2%
Fibrosis grade ≥ 2	7.4	9.1	11.4	14.3	15.3	79/802; 9.9%
Pain grade ≥ 2	3.2	3.7	3.7	4.5	4.5	28/812; 3.4%
Ulceration any grade	1.9	1.9	2.2	2.5	3.3	17/813; 2.1%
Hyperpigmentation ≥ 2	1.2	1.2	1.2	1.2	1.2	9/812; 1.1%
Breast edema grade ≥ 2	0.7	0.7	0.7	0.7	0.7	5/812; 0.6%
Retraction grade ≥ 2	0.3	0.3	0.3	0.3	0.3	2/812; 0.2%
Lymphedema grade ≥ 2	0	0	0	0	0	0/812; 0%

**Table 8:** Main retrospective analysis of toxicities after IORT boost with low-kV X-rays.

Author	Blank et al. [24]	Wenz et al. [25]	Pez et al. [22]	Wang et al. [23]
year	2010	2010	2019	2022
follow-up (median months)	37	34	78	64.8
patients (n)	197	154	400	451
IORT dose (Gy)	20	20	20	20
WBI dose (Gy)	46–50	46–50	46–50	-
fibrosis (≥II°)	37.9%	22.7%	19.1%	0.5%
teleangiectasia	12.1%	3.9%	10.0%	0.0%
hyperpigmentation (≥II°)	1.7%	0.6%	0.5%	1.3%
breast edema (≥II°)	0.0%	1.9%	2.4%	0.0%
lymph edema (≥II°)	0.0%	0.6%	0.0%	1.0%
retraction (≥II°)	0.0%	0.0%	-	0.0%
ulceration	0.0%	0.0%	0.3%	0.0%
pain (≥II°)	22.4%	0.6%	8.6%	0.0%

## Data Availability

The data presented in this study are available on request from the corresponding author due to privacy restrictions.
